# Comparative Research of Antioxidant, Antimicrobial, Antiprotozoal and Cytotoxic Activities of Edible *Suillus* sp. Fruiting Body Extracts

**DOI:** 10.3390/foods14071130

**Published:** 2025-03-25

**Authors:** Asta Judžentienė, Jonas Šarlauskas

**Affiliations:** 1 Institute of Biosciences, Life Sciences Center, Vilnius University, Saulėtekio Avenue 7, LT-10257 Vilnius, Lithuania; 2 Department of Organic Chemistry, Center for Physical Sciences and Technology, Saulėtekio Avenue 3, LT-10257 Vilnius, Lithuania; 3 Department of Xenobiotics Biochemistry, Institute of Biochemistry, Life Sciences Center, Vilnius University, LT-10257 Vilnius, Lithuania; jonas.sarlauskas@bchi.vu.lt

**Keywords:** *Suillus* sp., *Xerocomus* sp., *Tylopilus felleus*, *Phallus impudicus*, *Pycnoporus cinnabarinus*, antioxidant activity, gram-positive and gram-negative bacteria, fungi/yeasts, inhibition, in vitro, antiparasitic effects, cytotoxicity

## Abstract

The aim of this study was to evaluate bioactive properties of *Basidiomycota* fungi, mainly *Suillus* sp. Wide spectrum of activities were revealed for *S. variegatus*, *S. luteus*, *S. bovinus* and *S. granulatus;* and obtained results were compared with other common fungi. Total Phenolic Content (TPC) varied from 245.32 ± 5.45 to 580.77 ± 13.10 (mg (GAE) per 100 g of dry weight) in methanolic extracts of *S. bovinus* and *S. granulatus* fruiting bodies, respectively. In ethyl acetate extracts, the highest TPC were obtained for *S. variegatus* (310 ± 9.68, mg (GAE)/100 g, dry matter), and the lowest means for *S. luteus* (105 ± 3.55, mg (GAE)/100 g dry weight). The ethyl acetate extracts of the tested *Suillus* species exhibited a stronger antioxidant activity (AA) to scavenge DPPH^●^ and ABTS^•+^ than the methanolic ones, and the highest effects were determined for *S. luteus* (EC_50_, 0.15 ± 0.05 and 0.23 ± 0.05%, respectively). In the case of methanolic extracts, the highest AA were evaluated for *S. granulatus*. (EC_50_ for DPPH^●^ and ABTS^•+^, 0.81 ± 0.30 and 0.95 ± 0.22%, respectively). The ABTS^•+^ scavenging potential varied from 0.25 ± 0.05 to 0.74 ± 0.10 (mmol/L, TROLOX equivalent, for *S. granulatus* and *S. variegatus* fruiting body extracts, respectively) in the ethyl acetate extracts. *S. granulatus* extracts demonstrated the widest range of antimicrobial effects against both gram-positive and gram-negative bacteria (from 11.7 ± 1.3 to 28.5 ± 3.3 mm against *Pseudomonas aeruginosa* and *Bacillus mycoides*, respectively); and against two fungal strains (up to 13.6 ± 0.4 mm on *Meyerozyma guilliermondii*) in agar disc diffusion tests. Our study revealed that methanolic extracts of the most tested *Suillus* sp. were not active enough against the tested parasites: *Trypanosoma cruzi*, *Trypanosoma brucei*, *Leishmania infantum* and *Plasmodium falciparum*. Only *S. variegatus* extracts showed good antiprotozoal effects against *P. falciparum* (12.70 µg/mL). Cytotoxic activity was observed on human diploid lung cells MRC-5 SV2 by *S. granulatus* extracts (64.45 µg/mL). For comparative purposes, extracts of other common Lithuanian fungi, such as *Xerocomus* sp. (*X. badius*, *X. chrysenteron* and X. *subtomentosus*), *Tylopilus felleus*, *Phallus impudicus* and *Pycnoporus cinnabarinus* were investigated for their activity. The *P. cinnabarinus* extracts demonstrated the highest and broadest overall effects: 1.32 µg/mL against *T. brucei*, 1.46 µg/mL against *P. falciparum*, 3.93 µg/mL against *T. cruzi* and 21.53 µg/mL against *L. infantum*. Additionally, this extract exhibited strong cytotoxicity on MRC-5 cells (13.05 µg/mL). The investigation of bioactive fungal metabolites is important for the development of a new generation of antioxidants, antimicrobials, antiparasitic and anticancer agents.

## 1. Introduction

To date, it is estimated that approximately 150,000 species of fungi have been named and classified from its vast diversity, and even more, the annual number of new species descriptions added is currently around 2000 [1,2]. Of these, only 22,000 mushroom species have been described, and only a small percentage (up to 10%) has been the subject of scientific investigation [3].

*Suillus* is one of the most important genera of *Basidiomycota* fungi. To date, the morphological identification of *Suillus* genera has frequently been found to be misleading and needs to be investigated with more advanced techniques [4,5,6]. The majority of *Suillus* species are edible mushrooms that predominantly grow in conifer-dominated forests, primarily distributed in temperate regions of the Northern Hemisphere. The species are distinguished by their ability to generate a resistant spore bank, to achieve rapid colonization of roots, to establish positive biotic interactions with mammals, to create a long-distance dispersal capacity and to maintain a wide distribution [6]. Additionally, the ectomycorrhizal fungi could enhance the drought resistance of neighboring plants, especially in the *Pinus* woodlands [7,8,9]. However, mutualistic symbioses between certain trees and ectomycorrhizal fungi were revealed [10,11,12].

The *Suillus* mushrooms, the fruiting bodies of the fungi, are considered a significant source of bioactive compounds, such as amino acids, polysaccharides, proteins, phenolic acids, flavonoids, vitamins, natural pigments, steroids, fatty acids, triterpenoids and other (poly)phenolic derivatives [13,14,15,16,17,18,19,20,21,22,23,24,25,26,27,28,29,30,31,32,33]. The extracts derived from *Suillus* genus exhibited notable antioxidant [13,14,15,16,17,19,26,33], antimicrobial [13,16,19,22,23,30], anti-cancer [19,24,31], tyrosinase inhibitory activity [14,19], cytotoxic [17,32], anticholesteric [19], anti-HIV-1 activity [25] and immunostimulatory [18] properties. Edible *Suillus* sp. mushrooms represent a valuable food source and can also be applied as natural dietary supplements. Fruiting bodies are notable for their high fiber amount, low caloric and fat content, and are thus considered a valuable dietary component in the prevention of overweight. Furthermore, they are rich in proteins, vitamins (particularly the D and B group) and microelements, such as copper, selenium, potassium and others, which are essential for the proper functioning of the human heart, muscles and immune systems. Moreover, fungi may be used for therapeutic purposes or to reduce the risk of numerous human chronic diseases. Additionally, natural compounds with antimicrobial properties extracted from *Suillus* sp. could be applied for cosmetics applications [34].

Compared to the intensive pharmacological investigation and application of plant-derived natural compounds, only a small number of the most common species of macro-fungus have been investigated for biological activity [2,3,35]. The development of new drugs is urgently needed because of the new challenges posed by epidemics, continuous increase in cancer diseases, arising drug resistance and negative side effects associated with existing pharmaceuticals [30,35,36,37,38]. However, bioactive compounds could be isolated not only from mushrooms, but also from mushroom by-products, such as waste from fruiting bodies, mushrooms that do not comply with commercial standards, spent mushroom substrate from fermentation, mycelium and fermentation broth, etc. [39].

To the best of our knowledge, there is a definite lack of publications to date on the biological properties, such as in vitro antimicrobial, antioxidant, antileishmanial, antiprotozoal, antitrypanosomal and cytotoxic activity of edible *Suillus* sp. (*S. variegatus*, *S. luteus*, *S. bovinus* and *S. granulatus*) fruiting body extracts. Even more, there are no comparative studies on the biological activity of certain *Suillus* sp. with other fungi (*X. badius*, *X. chrysenteron*, *X. subtomentosus*, *T. felleus*, *P. impudicus* and *P. cinnabarinus*) growing in the same coniferous forest environment.

For these purposes, the main investigations were conducted. Firstly, the total phenolic content (TPC) in the extracts (in methanol/water (1:1, MeOH:H_2_O, *v*/*v*) and ethyl acetate) from fruiting bodies of four edible *Suillus* species, i.e., *S. variegatus*, *S. luteus*, *S. bovinus* and *S. granulatus* was determined; and antioxidant activity (AA) of different *Suillus* sp. extracts was evaluated by DPPH^•^ and ABTS^•+^ scavenging capacity. Secondly, the in vitro antimicrobial activity (AMA) of different *Suillus* sp. methanolic extracts against four gram-positive bacteria, such as *Staphylococcus aureus*, *Enterococcus faecalis*, *Bacillus mycoides* and *Bacillus cereus*, three gram-negative (*Pseudomonas aeruginosa*, *Escherichia coli* and *Proteus vulgaris*) bacteria and antifungal effects on four yeast/fungus (*Candida albicans*, *Rhodotorula rubra*, *Meyerozyma guilliermondii* and *Aspergillus niger*) strains by agar disc diffusion test was evaluated. Additionally, the activity of different *Suillus* sp. methanolic extracts against *S. aureus*, *E. coli* and *C. albicans* by serial dilution method was determined. Thirdly, the antileishmanial (ALA), antiprotozoal (APA) and cytotoxic activity (CA) of different *Suillus* sp. methanolic extracts against *Trypanosoma cruzi*, *Trypanosoma brucei*, *Leishmania infantum*, *Plasmodium falciparum* and on MRC-5 SV2 cell lines were revealed, respectively. Finally, the results obtained for *Suillus* sp. were compared with AMA (by serial dilution method), ALA, APA and CA of extracts from other species of mushrooms, such as *Xerocomus* sp. (*X. badius*, *X. chrysenteron* and *X. subtomentosus*), *Tylopilus felleus*, *Phallus impudicus* and *Picnoporus cinnabarinus*. In the search of novel antiparasitic compounds, the present study was specifically focused on mushroom extracts as a source of biologically active natural compounds, which may be superior to synthetic compounds because of their sufficiently low toxicity.

## 2. Materials and Methods

### 2.1. Material of Fruiting Bodies (Sporocarps) of the Mushrooms

The mushrooms of the *Suillus* genus, including *S. variegatus*, *S. luteus*, *S. bovinus* and *S. granulatus*; *Xerocomus* genus, including *X. badius*, *X. chrysenteron* and *X. subtomentosus*; *Tylopilus felleus*; *Phallus impudicus* and *Picnoporus cinnabarinus* were collected for the present study from the wild populations (spontaneously growing in the coniferous forests) in the Eastern South part of Lithuania. The identification of the mushrooms was carried out by Dr. D. Stankevičienė (Laboratory of Mycology, Institute of Botany, Nature Research Centre, Vilnius, Lithuania).

### 2.2. Preparation of Extracts from Fruiting Bodies by Different Solvents

Fruiting bodies (sporocarps) of the *Suillus* mushrooms were lyophilized and pulverized. The first extraction procedure was achieved with 10 g of mushroom material and 40 mL of a mixture of water and methanol (Merck KGaA, Darmstadt, Germany) (50:50, *v*:*v*) for 20 min, using stirring and centrifugation. The obtained extracts were filtered and concentrated in vacuo at 40 °C to dryness using a rotary evaporator. In order to calculate the yields, the obtained residues were weighed. Subsequently, the residues were resolved in a mixture of methanol and water (50:50, *v*/*v*), and these extracts were used for activity tests.

The second extraction procedure was conducted in the following manner: the remaining mushroom material after the first extraction was dried in a nitrogen flow and then extracted once more with 40 mL of ethyl acetate (Merck KGaA, Darmstadt, Germany) using a Soxhlet apparatus. After, the solvent was evaporated under vacuum, the residues were weighed (for yield calculation), and then re-diluted in ethyl acetate.

The obtained extracts were stored at −18 °C until bioactivity tests. Yields (%) of *Suillus* sp. in methanolic (MeOH:H_2_O, 50:50, *v*/*v*) and ethyl acetate extracts were determined (Table S1 in Supplementary Materials).

Extraction of *Xerocomus* sp., *Tylopilus felleus*, *Phallus impudicus* and *Picnoporus cinnabarinus* was performed using the methanol/water mixture (50:50) by the same first extraction method as used for *Suillus* sp. fruiting bodies.

### 2.3. Determination of Total Phenolic Content (TPC) in Different Suillus sp. Extracts

The determination of TPC was conducted in accordance with the standard Folin-Ciocalteu method that has been used in our laboratory [40]. The TPC results were expressed per weight of 100 g of dried fruiting bodies of the mushrooms. To 1 mL of Folin-Ciocalteu’s phenol reagent (Merck KGaA, Darmstadt, Germany), 0.5–1.0 mL of the studied mushroom body fruiting extracts (prepared according to the procedure described in Section 2.2) and 5 mL of double-distilled water were added, the contents were shaken and left for 5–8 min at room temperature. Then, 10 mL of Na_2_CO_3_ (Merck KGaA, Darmstadt, Germany; 7% *w*/*v*) was added; and the total volume was adjusted to 25 mL with bi-distilled water. The mixture was left in the darkness for 2 h (at 20–23 °C). The measurements (at 750 nm wavelength) were performed using a spectrophotometer (UV/Vis C-7200S, PEAK Instruments Inc.; Houston, TX, USA); and the obtained data were expressed as mg/L of gallic acid equivalent (GAE, Merck KGaA, Darmstadt, Germany), then re-calculated for 100 g of dry material.

### 2.4. Antioxidant Activity (AA) Tests by Spectrophotometric Methods, Using Antioxidant ABTS^•+^ Capacity and DPPH Radical Scavenging Assays

DPPH^•^ (2,2-diphenyl-1-picrylhydrazyl) and ABTS^•+^ (2,2′-amino-bis(ethylbenzothiazoline-6-sulfonic acid) diammonium salt) scavenging assays are reliable methods for the evaluation of the antioxidant capacity of biological substrates. The radical scavenging activity is generally expressed by the percentage of free radicals that are inhibited by antioxidants. The EC_50_ (concentration required to obtain a 50% antioxidant effect) is a parameter that is commonly employed to express the antioxidant capacity and to compare the activity of different compounds.

Antioxidant ABTS^•+^ capacity and TROLOX equivalent ABTS^•+^ assays were applied for various *Suillus* sp. (*S. variegatus*, *S. luteus*, *S. bovinus* and *S. granulatus*) methanolic (MeOH:H_2_O, 50:50, *v*/*v*) and ethyl acetate extracts according to the methods described in the literature [40]. Furthermore, the DPPH• assay was conducted for the aforementioned *Suillus* sp. extracts, employing the method that had been described previously [40].

### 2.5. Antibacterial and Antifungal Activity Assay

Testing of antibacterial activity was done by disc agar diffusion and by serial dilution methods [41]. Antibacterial activity of extracts from the fruiting bodies of mushrooms was tested against the above-mentioned gram-positive and gram-negative bacteria; and antifungal effects against yeasts/fungi. The bacteria were grown on Mueller-Hinton agar media (Merck KGaA, Darmstadt, Germany; pH 7.3). The agar media were poured into the plates to a uniform depth of 5 mm and allowed to solidify. The microbial suspensions at 5 × 10^6^ cfu/mL were streaked over the surface of media using a sterile Grigalski spatula to ensure confluent growth of the organism. The disc characteristics: 10 µL aliquots of the fruiting body extract 5.0% (*w*/*v*) in dimethyl sulfoxide (DMSO, Merck KGaA, Darmstadt, Germany) (1.15%), spotted on filter paper discs (Whatman No. 1, diameter 10 mm), which were then aseptically applied to the surface of agar plates at well-spaced intervals. The plates were incubated for 24–48 h (at +37 °C) and the observed growth inhibition zones, including disc diameters were measured. The fungi were cultured in Sabouraud’s agar (Liofilchem SRL, Roseto degli Abruzzi (TE), Italy) and suspensions at 5 × 10^6^ cfu/mL were applied. A quantity of 10 µL of the mushroom extract (5.0%, *w*/*v*) in DMSO, was impregnated on discs amplitude with 10 µL of DMSO and nystatin (2 µg per disc). Gram-positive bacteria *Staphylococcus aureus* BIGTC-BK06, *Bacillus mycoides* (DPB-Tc2 and DPKI-01), and *Bacillus cereus* DPKI-02; gram-negative bacteria *Escherichia coli* BIGTC-BK08 and *Pseudomonas aeruginosa* DPK-TcX; yeasts *Candida albicans* BIGTC-MK2, *Rhodotorula rubra* (DPm0454, DPm0423 and DPm0403) and *Aspergillus niger* Tiegh. DPF-Tc7 were obtained from the Microorganisms Culture Collection of Nature Research Centre (Vilnius, Lithuania).

### 2.6. Cytotoxicity Assay on MRC-5 Line Cells

The experimental procedure for the assessment of cytotoxic activity (CA) on MRC-5 SV2 cells was conducted in accordance with the method documented in the literature [42].

The MRC-5 SV2 cells, which were originally developed from a human diploid lung cell line, were cultivated in minimal essential medium (MEM, Thermo Fisher Scientific Inc., Waltham, MA, USA), with the following supplements: L-glutamine (20 mM), sodium hydrogen carbonate (16.5 mM), and 5% fetal calf serum (FCS). For the assay, 10^4^ MRC-5 cells/well were seeded onto the test plates containing the prediluted sample of mushroom extract; and subsequently incubated at 37 °C (5% CO_2_) for a period of 72 h. The assessment of cell viability was conducted using fluorimetric analysis following the incorporation of resazurin into the sample, after a 4-h period. Fluorescence intensity was measured (excitation at 550 nm, emission at 590 nm), and the results were expressed as a percentage of cell viability reduction compared to the control.

### 2.7. Anti-Trypanosomal Assays

The anti-trypanosomal activity tests described previously in the literature [42] were conducted using the cultured *Trypanosoma brucei* Squib-427 strain (suramin-sensitive).

*T. brucei brucei*, *T. cruzi* and *Leishmania infantum* bloodstream forms were received from Prof. L. Maes (University of Antwerp, Faculty of Pharmaceutical, Biomedical and Veterinary Sciences, Laboratory of Microbiology, Parasitology and Hygiene, Antwerp, Belgium). Reference materials (chloroquine, miltefosine, benznidazole, tamoxifen and suramin) were purchased from Sigma-Aldrich (Merck KGaA, Darmstadt, Germany).

### 2.8. Antileishmanian Assay

*Leishmania donovani* MHOM/ET/67/L82 species were maintained in the Golden Hamster, and spleen amastigotes were collected for preparing infection inocula. Primary peritoneal mouse macrophages were used as host cells; and they were collected after 2 days, after peritoneal stimulation with 2% potato starch suspension. Assays were performed in 96-well microtiter-plates, each well containing 10 µL of the mushroom extract dilutions together with 190 µL of macrophage/parasite inoculums (3 × 10^5^ cells + 3 × 10^6^ parasites/well/RPMI-1640 + 5% FCS). RPMI (Roswell Park Memorial Institute)1640 medium purchased from Thermo Fisher Scientific Inc. (Waltham, MA, USA) was applied in the assay. After 5 days incubation, parasite burdens (mean number of amastigotes/macrophage) are microscopically assessed after Giemsa staining. The results were expressed as % of reduction in parasite burden compared to untreated control wells, and IC_50_ (50% inhibitory concentration) was calculated.

### 2.9. Antiplasmodial Test

Chloroquine-resistant *P. falciparum* K1-strain was cultured in human erythrocytes O^+^ at +37 °C under a low oxygen atmosphere (93% N_2_, 4% CO_2_ and 3% O_2_) in RPMI-1640, supplemented with 10% human serum. Infected human red blood cells (200 µL, 1% parasitemia, 2% hematocrit) were added to each well of the plates with the tested mushroom extract and incubated for 72 h. After incubation, test plates were frozen at −20 °C. Parasite multiplication was measured using the Malstat assay, a colorimetric method based on the reduction of 3-acetyl pyridine adenine dinucleotide by parasite-specific lactate-dehydrogenase. Chloroquine diphosphate was utilized as an antiplasmodial reference drug. The results were expressed by % of reduction in parasitemia compared to control wells. The concentration causing 50% inhibition of parasite growth (IC_50_) was calculated from the drug concentration—response curves. Each assay was conducted in triplicate. The K1strain of *P. falciparum* was received from Prof L. Maes of the Laboratory of Microbiology, Parasitology and Hygiene, Faculty of Pharmaceutical Sciences, Biomedical and Veterinary Sciences of the University of Antwerp, Belgium.

### 2.10. Statistical Analysis

The obtained results were statistically processed using the XLSTAT programme (Premium, trial version, Addinsoft 2014, Paris, France); and the data were presented as means with standard deviation (SD) values. Statistically significant differences were revealed by One-way ANOVA statistical analysis between tested parameters for each *Suillus* species. Differences were compared using Student’s *t*-test, at a significance level of *α* = 0.05. *p*-Values were calculated by IBM SPSS Statistics software (Version 28.0.1.1(15), New York, NY, USA); and statistically significant differences were set at *p* ≤ 0.05.

## 3. Results and Discussion

### 3.1. Total Phenolic Content (TPC) in the Extracts from Fruiting Bodies of the Suillus *sp.* Mushrooms

The total amount of phenolic compounds varied greatly depending on applied solvent and on the *Suillus* species (Table 1). In the methanolic extracts, the lowest TPC was determined for *S. bovinus* (average 248.32 mg, expressed by GAE in 100 g of dry weight), while in *S. granulatus,* the content was almost 2-fold bigger (580.77 mg/100 g). In the case of secondary extraction by ethyl acetate, the lowest quantity of phenols was measured for *S. luteus* (average 104.90 mg (GAE) in 100 g of dry material), and approximately 3-fold higher levels were found in *S. variegatus* extracts (310.03 mg/100 g of dry matter). In comparison with the results obtained in this study, high levels of organic acids were determined in the extracts (prepared by boiling in water for 30 min) of *S. luteus* (ca. 34–67 g/kg dry matter) and *S. granulatus* (ca. 10–167 g/kg) collected in northeastern Portugal [15]. Pereira et al. [33] found 58 mg of polyphenols (expressed as GAE) and 33 mg of flavonoids (as (+)-catechin equivalent) in 1 g of the Portuguese *S. variegatus* methanolic extract. However, an investigation of Polish *S. luteus* [16] revealed the presence of approximately 8.2 g of total polyphenols (expressed as GAE) and 3.7 g of flavonoids (as (+)-catechin equivalent) per kg of dry weight of the mushrooms.

### 3.2. Results of Antioxidant Activity (AA) of the Suillus *sp.* Extracts

The AA of the *Suillus* sp. extracts was evaluated by spectrophotometric DPPH^●^ scavenging test. The data on scavenging potential (50% of DPPH free radicals, EC_50_, %) of various *Suillus* sp. extracts were presented in Table S2 (Supplementary Materials).

**Figure 1:**
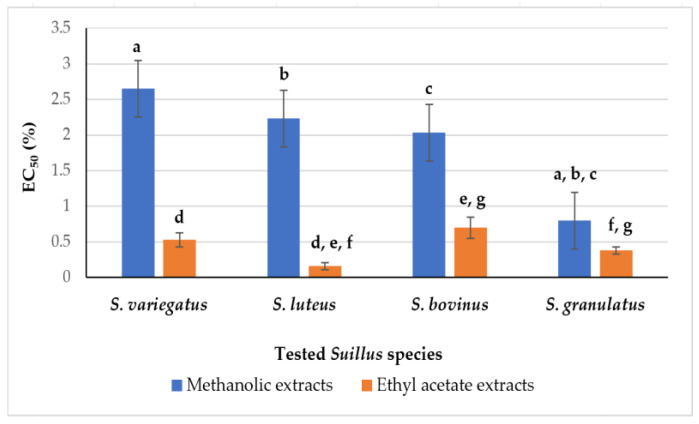
Scavenging potential: effective concentration (EC_50_, %) of various *Suillus* sp. extracts capable to scavenge 50% of DPPH^●^. Data with significant differences (*p* < 0.05) are indicated by the letters: a, b, c for methanolic; and d, e, f, g for ethyl acetate solutions, respectively.

In the case of methanolic *Suillus* sp. extracts (MeOH:H_2_O, 50:50, *v*:*v*), the strongest antioxidant properties, presenting the lowest EC_50_ values (EC_50_ = 0.80 ± 0.30%) were evaluated for *S. granulatus* fruiting bodies (Figure 1).

No significant differences were determined between methanolic extracts of other species (*S. variegatus*, *S. luteus* and *S. bovinus*), where EC_50_ values change from 2.03 ± 0.40 to 2.65 ± 0.36%. Only the AA results obtained for *S. granulatus* methanolic extracts correlated very well with TPC in them (Table 1).

The ethyl acetate extracts of the tested *Suillus* species exhibited a stronger AA than the methanolic ones. The highest activity was determined for *S. luteus* (EC_50_ = 0.16 ± 0.05%), while *S. bovinus* extracts exhibited the weakest effects (EC_50_ = 0.70 ± 0.15%) in ethyl acetate solutions (Figure 1). The obtained results did not correlate precisely with TPC data (Table 1), and it could be explained that not only phenolic compounds but also other constituents more soluble in ethyl acetate than in methanol/water were responsible for the scavenging activity of DPPH radicals.

**Figure 2:**
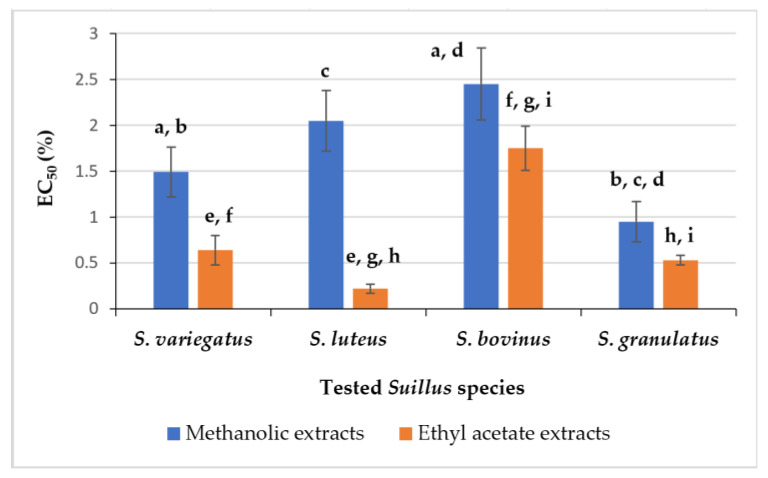
Scavenging potential: effective concentration (EC_50_, %) of various *Suillus* sp. extracts capable to scavenge 50% of ABTS^•+^. Data with significant differences (*p* < 0.05) are indicated by the letters: a, b, c and d for methanolic; and e, f, g, h and i for ethyl acetate extracts, respectively.

Additionally, the AA of the *Suillus* sp. extracts was assessed by spectrophotometric ABTS^•+^ scavenging test. Data of scavenging activity of 50% ABTS^•+^ (EC_50_, %) by various *Suillus* sp. extracts were presented in Table S3 (Supplementary Materials). In the case of methanolic *Suillus* sp. extracts, the strongest ability to scavenge free radicals of ABTS cations, and expressing by the lowest EC_50_ values (EC_50_ = 0.95 ± 0.22%) was revealed for *S. granulatus* fruiting bodies (Figure 2).

For the rest of the species, the values of effective concentration varied from 1.49 ± 0.27 to 2.45 ± 0.39%; and significant differences were observed between the AA of *S. variegatus* and *S. bovinus* extracts. In the case of extracts prepared in ethyl acetate, the strongest AA was determined for *S. luteus* fruiting bodies (EC_50_ = 0.22 ± 0.05%). The extracts of *S. bovinus* exhibited the weakest effects (EC_50_ = 1.75 ± 0.24%) (Figure 2). The results from this method correlated very well with the data obtained by DPPH free radicals scavenging activity assay (Figure 1).

Methanolic extracts of tested *Suillus* sp. demonstrated slightly different activity to scavenge DPPH^●^ and ABTS^•+^, while the solutions prepared in ethyl acetate showed the same scavenging capacity according to the tested species. The order of the scavenging potential of both radicals of *Suillus* sp. in ethyl acetate was as follows: *S. luteus* > *S. granulatus* > *S. variegatus* > *S. bovinus*.

**Figure 3:**
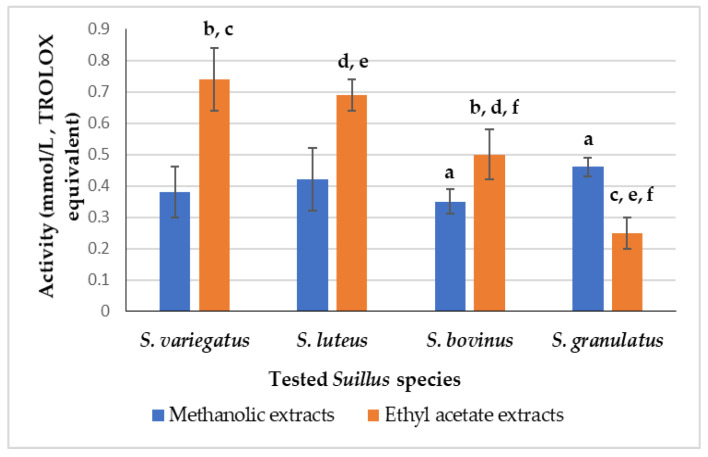
ABTS^•+^ scavenging activity (mmol/L, expressed by TROLOX equivalent) of various *Suillus* sp. extracts. Means with statistically significant differences (*p* < 0.05) are marked with the letters: a for methanolic; and b, c, d, e, f for ethyl acetate solutions, respectively.

The results of ABTS^•+^ scavenging capacity were expressed also by TROLOX equivalent (Table S4 in Supplementary Materials; and Figure 3).

Significant differences were revealed only between *S. bovinus* and *S. granulatus* methanolic extracts (0.35 ± 0.04 and 0.47 ± 0.03 mmol/L, respectively). In the case of extracts prepared in ethyl acetate, the ABTS^•+^ scavenging activity varied from 0.26 ± 0.05 to 0.73 ± 0.10 (mmol/L, TROLOX equivalent, for *S. granulatus* and *S. variegatus* fruiting body extracts, respectively).

A comparison of the AA of *Suillus* sp. extracts with the literature data is complicated by the fact that the results were obtained by various techniques and are expressed in different ways. In comparison to other species of mushroom, *Salicaceae*, in general, exhibits moderate antioxidant activity [15,16,33]. In agreement with our research, the reducing power of DPPH^•^ was stronger for *S. granulatus* than for *S. luteus*, the wild mushrooms from Portugal [15]. Newly discovered polyprenylphenol derivatives from *S. luteus* exhibited significant DPPH^•^ scavenging activity with IC_50_ values ranging from 1.55 ± 0.29 to 19.89 ± 2.28 μM [14]. The AA of *S. luteus* methanolic extracts tested by Jaworska et al. [16] by various methods, such as DPPH free radical and ABTS radical cation scavenging, as well as by the FRAP method, showed moderate effects (7.78 and 3.48 mM TROLOX equivalent for DPPH^•^ and ABTS^•+^ respectively, and using the FRAP method, it was 9.15 mM Fe^2+^). In addition, *Suillus* sp. (the species name is not specified) extracts exhibited a remarkable AA by reducing Fe^+3^ to Fe^+2^ ions in another study [13]. Aytar et al. [43] revealed that *S. luteus* extracts had more potent free radical scavenging activity than standard antioxidants BHT (extract in methanol (IC_50_, 63.72 μg/mL) > extract in ethanol (IC_50_, 80.72 μg/mL) > BHT (IC_50_, 96.47 μg/mL)). However, AA of *S. granulatus* was determined by using five complementary tests: *β*-carotene-linoleic acid, DPPH^•^ and ABTS^•+^ scavenging, metal chelating and CUPRAC assays [26]. In DPPH^•^ scavenging, ABTS^•+^ scavenging and CUPRAC assays, the ethyl acetate extract of *S. granulatus* showed the best activity 91.52 ± 0.97%, 89.67 ± 0.15% and 3.90 ± 0.09 at 400 μg/mL concentration, respectively, while in *β*-carotene-linoleic acid assay, the methanol extract of this mushroom exhibited higher activity in the above study. Our obtained results also showed the same tendency: *Suillus* sp. extracts in ethyl acetate were more active than the methanolic ones. Also, comparable AA values were obtained for *S. granulatus* (growing in Brazil) ethanolic extract (507.61 µM TROLOX/g) [44] and in the present study.

### 3.3. Antimicrobial Activity (AMA) of the Suillus sp. Fruiting Bodies Extracts in Agar Disc Diffusion Test

Methanolic (MeOH:H_2_O, 50:50, *v*/*v*) extracts obtained from fruiting bodies of four *Suillus* species (*S. variegatus*, *S. luteus*, *S. bovinus* and *S. granulatus*) were tested against four gram-positive (*Staphylococcus aureus*, *Enterococcus faecalis*, *Bacillus mycoides* and *Bacillus cereus*) bacteria, three gram-negative (*Pseudomonas aeruginosa*, *Escherichia coli* and *Proteus vulgaris*) bacteria, and four yeast/fungus (*Candida albicans*, *Rhodotorula rubra*, *Meyerozyma guilliermondii* and *Aspergillus niger*) strains (Table 2). Only methanolic extracts containing 2.5 and 5% were applied for the AMA test. Fruiting body extracts prepared with ethyl acetate were not investigated by agar disc diffusion test because of the reason that they contained a large amount of insoluble, highly lipophilic constituents. Of all investigated species, *S. granulatus* fruiting body extracts demonstrated a widest range of antimicrobial effects against both gram-positive and gram-negative bacteria; and against two fungal strains. The extracts exhibited the strongest activity against *Staphylococcus aureus*. Solutions of 2.5 and 5% of *S. variegatus* extracts exhibited strong inhibitory effects on *Enterococcus faecalis* and all tested pathogenic fungi (except *Aspergillus niger*); but no AMA were observed on bacteria *Staphylococcus aureus*, *Bacillus mycoides* and *Escherichia coli*. Solutions of 5% *S. luteus* extracts demonstrated AMA against all tested gram-positive (except *Enterococcus faecalis*) and negative bacteria; and two fungal (*Candida albicans* and *Rhodotorula rubra*) strains. Among all tested *Suillus* sp., the extracts of *S. luteus* showed the strongest inhibition effects against *Bacillus mycoides*. Extracts of *S. bovinus* exhibited the strongest AMA against *E. coli*.

To compare the AMA of *Suillus* sp. extracts with literature data is complicated because of the lack of studies, and the results were obtained by using different methods and various strains of microorganisms. Aytar et al. reported that various *S. luteus* extracts had a relatively low AMA against numerous bacterial (*S. aureus*, *B. cereus*, *B. subtilis*, *E. coli*, *E. faecalis*, *E. hirae*, *K. pneumoniae*, *P. aeruginosa*, *P. vulgaris*, *S. typhimurium*) and fungal strains (*C. tropicalis*, *C. albicans* and *C. krusei*), as determined by agar well method [43]. The activity of *S. granulatus* ethanolic extracts tested by Volcão et al. against *P. aeruginosa* was moderate (MIC = 5 mg/mL and IC_90_ = 1.814 mg/mL) [44]. As demonstrated in another study [13], the *b*-carotene isolated from *Suillus* sp. (for which no species name is provided) exhibited a significant AMA at a concentration of 100 mg/mL against *K. pneumonia* (bacterial growth inhibition was 40 mm), *E. coli* (36 mm) and *S. aureus* (31 mm); and no effects were observed against *P. aeruginosa*.

### 3.4. Results of Cytotoxic, Antiprotozoal, Antitrypanosomal, Antileishmanial and Antimicrobial Activity of Mushroom Extracts

To date, bioactive compounds of *Ectomycorrhiza* fungi with antiprotozoal, nematicidal or insecticidal activities have not been sufficiently investigated. Our study revealed that methanolic extracts (MeOH:H_2_O, 50:50, *v*/*v*) of the most tested *Suillus* sp. were not enough active against tested parasites, such as *Trypanosoma cruzi*, *Trypanosoma brucei*, *Leishmania infantum* and *Plasmodium falciparum*; and against some bacteria (*Candida albicans*, *Staphylococcus aureus* and *Escherichia coli*, by series dilution method) (Table 3). Only *S. variegatus* extracts showed antiprotozoal effects against *P. falciparum* (12.70 µg/mL). To the best of our knowledge, data on antiprotozoal, antitrypanosomal and antileishmanial activity of the *Suillus* sp. (*S. variegatus*, *S. luteus*, *S. bovinus* and *S. granulatus*) extracts were presented for the first time.

A weak cytotoxic activity was observed on human diploid lung cells MRC-5 SV2 by *S. granulatus* extracts (64.45 µg/mL) under this study. The literature survey revealed that various extracts of *Suillus* sp. or individual compounds isolated from these mushrooms can have anticancer properties. According to Stojanova et al. [17], *S. granulatus* extracts containing *α*- and *β*-glucans, demonstrated moderate antitumor activity against the cervical cancer cell line HeLa and against the human hepatocellular carcinoma cell line HepG2. However, the anti-inflammatory and immunomodulatory effects of *β*-glucans from edible mushrooms were evaluated [45,46]. The compound suillin, isolated by dichloromethane from *S. granulatus* was active against human rhino-pharyngeal cancer (KB), murine leukemia (*p*-388) and human bronchopulmonary carcinoma (NSCLC-N6) cell lines [47]. Six newly discovered polyphenolic metabolites from the fungus *S. granulatus* were active against the HepG2 liver cancer cell line; and suillusol B was identified as the most active compound [32]. *S. granulatus* and *S. luteus* methanolic extracts exhibited a significant cytotoxic activity against two murine cancer cell lines, L1210 and 3LL [48]. The methanolic extracts of *S. luteus* demonstrated notable antitumor activity on the breast cancer cell line MCF-7 (IC_50_ ≈ 173 µg/mL) [49]. However, various *S. luteus* extracts were found to be active against the lung cancer NCI-H460 (GI_50_ = 30.33 µg/mL), the gastric cancer AGS (GI_50_ = 30.30 µg/mL), the breast cancer MCF-7 (GI_50_ = 32.75 µg/mL) and the colon cancer HCT-15 cells (GI_50_ = 17.75 µg/mL) [49,50]. A new phytosphingosine-type ceramide, suillumide, isolated from the EtOH extract of the basidiomycete *S. luteus* showed cytotoxic effect against the human melanoma cell line SK-MEL-1 [51]. The steroidal derivatives, ergosterol and its peroxide, present in the extracts of a number of edible mushrooms possess anticancer properties [52].

Additionally, for comparative purposes, other species of common Lithuanian mushrooms, such as *Xerocomus* sp. (*X. badius*, *X. chrysenteron* and *X. subtomentosus*), *Tylopilus felleus*, *Phallus impudicus* and *Pycnoporus cinnabarinus* were selected for the activity tests. Some of them are edible (e.g., *Xerocomus* sp.), some are commonly inedible by humans but are a food for wild animals (e.g., *T. felleus*). *T. felleus*, commonly known as the bitter bolete or the bitter tylopilus, is the only species found in Europe. *T. felleus* has a bitter taste, and due to the unpleasant smell and taste, it is generally considered as inedible fungi. However, *T. felleus* is not a toxic mushroom; and currently is included to the permitted mushrooms list for human consumption in many countries [53,54]. In Lithuania, the dried pulverized form of the bitter bolete is utilised as a distinctive pepper substitute or for flavouring purposes.

*P. impudicus* is a widespread fungus with a phallic shape when mature and foul odor, but it is not considered a poisonous mushroom. Dimethyl oligosulfides are the most significant contributors to the strong and foul odor of stinkhorn fruit bodies [55,56]. Despite its unpleasant smell, immature *P. impudicus* mushrooms are consumed in some European (Germany, France, Lithuania, the Czech Republic) and Asian (China) countries; even the fungus is a commercially available product [56]. The fungus is known under the names Earth’s fat or Wolf’s mushroom in Lithuanian. It has been documented that *P. impudicus* is applied as a functional foodstuff in Lithuania [57]. In Lithuanian cuisine, stinkhorn is consumed in some culinary preparations, including fresh (sliced and placed on bread) and cooked forms. Moreover, the fermented juice derived from *P. impudicus* is utilised as a dietary supplement in Lithuania and Latvia. A gelatinous (polysaccharide) fraction from immature *P. impudicus* is used as a folk remedy, which is applied as a natural cream-like material for cosmetic purposes (for skin hydration and possible rejuvenation effects) [58]. An alcoholic extract of this mushroom is employed as a remedy in folk medicine for the treatment of rheumatic diseases and podagra.

*P. cinnabarinus* is a saprophytic fungus growing on hardwood trees, including beeches, birches and cherries, and decomposing wood. The red pigments obtained from two *Pycnoporus* sp., *P. cinnabarinus* and *P. sanguineus*, were suggested as functional additives and color enhancers instead of synthetic colorants for their application in food and cosmetic industry [59]. Moreover, phenoxazine-type colored compounds from *P. cinnabarinus* were applied as functional pigments in food processing [60].

Lithuania is a mycophilic country with long-standing traditions of mushroom foraging and usage. These practices encompass both the use of mushrooms as food and their application in ethno-pharmacology [58]. Data of cytotoxic, antiprotozoal, antitrypanosomal, antileishmanian and antimicrobial properties of totally 10 macromycete extracts were presented in Table 3. The *P. cinnabarinus* extracts demonstrated the highest and broadest overall biological effects, starting from 1.32 µg/mL against *T. brucei*, 1.46 µg/mL against *P. falciparum* and 21.53 µg/mL against *L. infantum*. Additionally, this extract revealed strong cytotoxicity (13.05 µg/mL) on MRC-5 cell lines, antiprotozoal effects on *T. cruzi* (3.93 µg/mL) and weak antibacterial activity against *S. aureus* pathogen (69.71 µg/mL). It has been reported that the fungus *P. cinnabarinus* is able to bio-synthesize bioactive compounds, such as alkaloids (e.g., pycnoporin), pigments (cinnabarin, cinnabarinic acid, tramesanguin, phlebiarubrone), steroid derivatives (e.g., ergosterol peroxide) and other constituents with antitumor, antimicrobial and anti-inflammatory activities [60,61,62,63,64,65,66,67]. The number of studies related to antiprotozoal, antitrypanosomal, antileishmanial or anti-plasmodial activity of *P. cinnabarinus* extracts is very limited.

Methanolic extracts of *X. badius* and *X. subtomentosus* fruiting bodies showed a weak antiprotozoal activity against *T. cruzi* (71.01 and 72.33 µg/mL, respectively); and no cytotoxicity on MRC-5 cell lines. *X. chrysenteron* extracts demonstrated good activity against *T. cruzi* and *T. brucei* trypanosome parasites (17.59 and 16.92 µg/mL, respectively). In the antibacterial assay, a weak activity was found against *S. aureus* for *X. chrysenteron* extracts (84.94 µg/mL). The edible mushroom *X. chrysenteron* possesses insecticidal properties, and some proteins are responsible for its strong toxicity to some insects (such as the dipteran *Drosophila melanogaster* and the hemipteran *Acyrthosiphon pisum*) [68]. Also, it was revealed that various extracts of *Xerocomus* sp., containing mostly carbohydrates, proteins, flavonoids, fatty acids, B-group vitamins, tocopherols, phenolic acids, carotenoids, pigments and minerals, demonstrated strong antioxidant properties [27,68,69,70,71,72,73,74].

Our investigated methanolic extracts of *T. felleus* exhibited a strong antiprotozoal activity (against *T. cruzi*, 5.54 µg/mL) and weak cytotoxicity (on human diploid lung cells MRC-5 SV2, 64.45 µg/mL) (Table 3). Only few studies about biological activities of *T. felleus* extracts or their individual constituents have been performed [75,76,77,78,79]. The cytotoxicity tests of *T. felleus* extracts, prepared in ethanol and chloroform, showed a selective significant activity against cancer cells MCF-7 [75]. Antibacterial effects of *T. felleus* extract were established against *Salmonella infantis* strain in the research of Sterniša et al. [76], however, anti-biofilm activity was not detected against this bacteria strain in the study. The antitumor activity of tylopilan, a glucan isolated from the fruiting bodies of *T. felleus*, was demonstrated against 180-TG Crocker tumor cells in vitro [77]. Moreover, bone regeneration, anti-diabetic, cholesterol and blood pressure lowering, immunomodulating and other effects of β-glucans were estimated [78].

Many species of wild growing edible mushrooms are valuable sources of nutrients and mineral components, which are indispensable for human health [25,67,70,80]. On the other hand, it should be mentioned that some mushrooms, e.g., *X. badius*, *X. chrysenteron*, *X. subtomentosus*, *T. felleus* and *Suillus* sp. tend to bio-accumulate traces of metals (including hazardous) and radionuclides [27,74,81,82,83,84,85,86,87,88,89,90,91].

*P. impudicus* methanolic extracts did not demonstrate any remarkable activity under the study, while antioxidant, cytotoxic, immunomodulating, wound healing properties and hypoglycemic potential were revealed for the common stinkhorn extracts [52,92,93,94,95].

## 4. Conclusions

For the first time, a comparative study of the biological properties of edible *Suillus* sp. (*S. variegatus*, *S. luteus*, *S. bovinus* and *S. granulatus*) was conducted.

The total phenolic content (TPC) and antioxidant activity (AA) of four *Suillus* species in different extracts (in methanol/water and ethyl acetate) were measured and compared. The ethyl acetate extracts of the tested fruiting bodies of *Suillus* sp. exhibited a stronger AA than the methanolic ones, and the highest capacity of DPPH^●^ scavenging was revealed for *S. luteus* (EC_50_ = 0.16 ± 0.05%). In comparison to *Suillus* sp., the highest TPC (580.77 ± 13.10 mg GAE/100 g, dry matter), and the strongest AA to scavenge DPPH^●^ (EC_50_ = 0.80 ± 0.30%) were evaluated for *S. granulatus* methanolic extracts. Methanolic extracts of tested *Suillus* sp. demonstrated slightly different activity to scavenge DPPH^●^ and ABTS^•+^, while the solutions prepared in ethyl acetate showed the same scavenging tendency according to the tested species.

*S. granulatus* fruiting body extracts demonstrated a widest range of antimicrobial activity (AMA) against both gram-positive (inhibition zone was up to 28.5 ± 3.3 mm) and gram-negative bacteria (≤18.4 ± 0.6 mm); and antifungal activity against two fungal strains (≤13.6 ± 0.4 mm). The extracts of *S. variegatus* exhibited strong inhibitory effects on *Enterococcus faecalis* (inhibition zone ≤ 40.6 ± 4.4 mm) and on all tested pathogenic fungi (except *Aspergillus niger*); but no AMA were observed on bacteria *Staphylococcus aureus*, *Bacillus mycoides* and *Escherichia coli*. The *S. luteus* extracts showed the strongest inhibition effects against *B. mycoides* (up to 30.2 ± 1.8 mm), while extracts of *S. bovinus* exhibited the strongest AMA against *E. coli* (≤22.3 ± 3.6 mm). All tested *Suillus* sp. extracts were totally inactive against *A. niger* fungus.

A comparison of the various biological properties (antiprotozoal, antibacterial and antifungal) of *Suillus* sp. with other common Lithuanian mushrooms, such as *Xerocomus* sp. (*X. badius*, *X. chrysenteron* and *X. subtomentosus*), *Tylopilus felleus*, *Phallus impudicus* and *Pycnoporus cinnabarinus*, was conducted, and significant differences were revealed. Tested *Suillus* sp. extracts did not exhibit AMA against some bacteria (*Candida albicans*, *Staphylococcus aureus* and *Escherichia coli*) using the serial dilution method. A selective antiplasmodial activity (against *Plasmodium falciparum*, IC_50_ = 12.7 µg/mL) was revealed by *S. variegatus* extract. Five mushroom extracts (*Xerocomus* sp.; *T. felleus* and *P. cinnabarinus*) demonstrated activity against protozoal parasites (*Trypanosoma cruzi*), but unfortunately one of them, the most active, *P. cinnabarinus* (IC_50_ = 3.93 µg/mL), was also toxic on MRC-5 cells (CC_50_ = 13.05 µg/mL). *T. felleus* also demonstrated strong effects against *T. cruzi* (IC_50_ = 5.54 µg/mL). *X. chrysenteron* was selectively active against *T. brucei* (IC_50_ = 16.92 µg/mL). *P. cinnabarinus* showed a moderate but not specific activity against *Leishmania infantum* (IC_50_ = 21.53 µg/mL).

The present research demonstrated that *Basidiomycota* fungi can be an important source for the discovery of new natural compounds with various medicinal properties, especially targeting inflectional and parasitic diseases. In forthcoming studies, the primary objectives will be the bioassay-guided fractionation, separation and isolation of active compounds and/or fractions from mushrooms that are responsible for antioxidant, antimicrobial or antiparasitic activity.

## Figures and Tables

**Table 1:** TPC (mg GAE/100 g of dry matter; values were expressed as means ± SD (n = 3)) in methanolic (1:1, MeOH:H_2_O, *v*/*v*) and ethyl acetate extracts of different *Suillus* species.

*Suillus* Species	Methanol/Water	Ethyl Acetate
*S. variegatus*	340.11 ± 8.98	310.03 ± 9.68
*S. luteus*	393.47 ± 9.83	104.90 ± 3.55
*S. bovinus*	248.32 ± 5.45	178.22 ± 4.12
*S. granulatus*	580.77 ± 13.10	250.15 ± 6.34

**Table 2:** Mean diameters of inhibition zones (mm, expressed as means ± SD (n = 3)) of the microorganisms using fruiting body extracts of *Suillus* genus in the agar disc diffusion test *.

Extracts and Standards		Tested Bacteria and Fungi										
		*S. aureus*	*E. faecalis*	*B. mycoides*	*B. cereus*	*P. aeruginosa*	*E. coli*	*P. vulgaris*	*C. albicans*	*Rh. rubra*	*M. guill.*	*A. niger*
*S. variegatus*	a	0 (C)	40.6 ± 4.4	0	10.3 ± 0.4	18.2 ± 3.1	C	12.1 ± 0.3	12.5 ± 1.3	11.7 ± 2.3	10.5 ± 0.2	0
	b	0	18.1 ± 2.7	0	10.4 ± 0.2	13.6 ± 2.4	0	12.1± 0.5	11.6 ± 1.3	10.7 ± 1.3	C	0
*S. luteus*	a	15.2 ± 3.7	0	30.2 ± 1.8	18.0 ± 1.4	15.6 ± 1.4	13.1 ± 1.0	14.6 ± 1.1	16.1 ± 0.9	12.5 ± 0.6	C	0
	b	C	0	25.4 ± 2.2	12.3 ± 0.7	14.6 ± 1.4	12.3 ± 0.7	12.0 ± 0.6	13.3 ± 2.2	12.5 ± 0.5	C	0
*S. bovinus*	a	0	0 (C)	0	11.4 ± 0.3	15.7± 2.3	22.3 ± 3.6	17.7± 2.5	16.3 ± 2.8	12.5 ± 0.5	14.3 ± 2.5	0
	b	0	0	0	10.5 ± 0.6	13.1 ± 0.9	16.1 ± 1.4	13.4 ± 1.7	12.0 ± 1.1	11.9 ± 1.0	11.0 ± 0.4	0
*S. granulatus*	a	24.7 ± 3.3	24.4 ± 2.4	28.5 ± 3.3	18.3 ± 1.8	11.7 ± 1.3	18.4 ± 0.6	16.1 ± 0.4	13.3 ± 1.4	C	13.6 ± 0.4	0
	b	14.3 ± 1.7	9.3 ± 1.4	23.0 ± 0.4	14.9 ± 0.7	9.9 ± 0.3	10.0 ± 1.0	12.6 ± 0.5	12.1 ± 0.6	C	12.1 ± 0.6	0
Chloramphenicol		24.5 ± 0.5	14.8 ± 1.2	28.2 ± 3.2	26.4 ± 1.2	20.2 ± 1.8	13.6 ± 1.4	32.5 ± 1.2				
Tetracycline		16.4 ± 15.2	11.8 ± 1.8	20.0 ± 2.2	19.6 ± 0.7	17.7 ± 2.2	17.7± 2.4	27.6 ± 1.7				
Nystatin									25.0 ± 2.8	16.7 ± 2.0	24.4 ± 3.2	31.1 ± 2.0

* The results were presented as means ± SD (Standard Deviation) of three replicate tests from three experiments (n = 9), paper of disc diameter was included. Solvent methanol as negative control was presented only at the highest concentration (10 μL/disc). Reference compounds were tetracycline (30 μg/disc) and chloramphenicol (2 µg/disc) for bacteria; and nystatine (30 μg/disc) for fungi. a—Extracts concentration was 5.0% (1.15% DMSO, as a control); b—extracts concentration was 2.5% (control—0.55% DMSO); C—impact of coated disc on activity, which could be influenced by volatile constituents (terpenoids, etc.).

**Table 3:** Results (IC_50_, expressed as µg/mL) of antiprotozoal (against *Trypanosoma cruzi*, *Trypanosoma brucei*, *Leishmania infantum* and *Plasmodium* falciparum); cytotoxic (on MRC-5 cells) and antimicrobial (against *Candida albicans*, *Staphylococcus aureus* and *Escherichia coli* by series dilution test) activity of mushroom extracts in vitro *.

Extract	MRC-5	*T. cruzi*	*T. brucei*	*L. infantum*	*P. falciparum*	*C. albicans*	*S. aureus*	*E. coli*
*S. variegatus*	>128	>128	>128	>128	12.70	>128	>128	>128
*S. luteus*	>128	>128	>128	>128	>128	>128	>128	>128
*S. bovinus*	>128	>128	>128	>128	>128	>128	>128	>128
*S. granulatus*	64.45	>128	>128	>128	>128	>128	>128	>128
*X. badius*	>128	71.01	>128	>128	>128	>128	>128	>128
*X. chrysenteron*	64.45	17.59	16.92	>128	>128	>128	84.94	>128
*X. subtomentosus*	>128	72.33	>128	>128	>128	>128	>128	>128
*T. felleus*	64.45	5.54	>128	>128	>128	>128	>128	>128
*P. impudicus*	>128	>128	>128	>128	>128	>128	>128	>128
*P. cinnabarinus*	13.05	3.93	1.32	21.53	1.46	>128	69.71	>128
Tamoxifen	10.5							
Benznidazole		2.65						
Suramin			0.03					
Miltefosine				3.3				
Chloroquine					0.3			

* The results were presented as means of two or three individual experiments. The following criteria were used to evaluate the level of extracts activity: IC_50_ ≤ 5 µg/mL—pronounced activity, 5 < IC_50_ < 20—good activity, 20 < IC_50_ < 30—moderate activity, 30 < IC_50_ < 80–low activity and IC_50_ > 128 µg/mL—as inactive. Samples were considered cytotoxic when CC_50_ < 32 µg/mL. References of pharmaceutical products were used in concentrations from 0.1 to 12 µg/mL.

## Data Availability

The original contributions presented in this study are included in the article/Supplementary Materials. Further inquiries can be directed to the corresponding author.

## Supplementary Materials

The following supporting information can be downloaded at: https://www.mdpi.com/article/10.3390/foods14071130/s1, Table S1: Yields (%) obtained from *Suillus* sp. fruiting bodies by first (using methanol/water (1:1)) and second (with ethyl acetate) extraction procedures. Table S2: Effective concentration (EC_50_, %) of various *Suillus* sp. extracts capable to scavenge 50% of DPPH^●^. Table S3: Effective concentration (EC_50_, %) of various *Suillus* sp. extracts capable to scavenge 50% of ABTS^•+^. Table S4: ABTS^•+^ scavenging activity (mmol/L, expressed by TROLOX equivalent) of various *Suillus* sp. extracts.

## Abbreviations

The following abbreviations are used in this manuscript:

TPC	Total Phenolic Content
AA	Antioxidant activity
GAE	Gallic acid equivalent
AMA	Antimicrobial activity
ALA	Antileishmanial activity
APA	Antiprotozoal activity
CA	Cytotoxic activity
AMA	Antimicrobial activity
RPMI	Cell culture medium
