# Supplementary material for: Comparative Research of Antioxidant, Antimicrobial, Antiprotozoal and Cytotoxic Activities of Edible Suillus sp. Fruiting Body Extracts
Source: Foods. 2025 Mar 25;14(7):1130. doi: 10.3390/foods14071130 (PMC11988316; doi:10.3390/foods14071130)
Supplement: Supplementary file 1 [file foods-14-01130-s001.zip › foods-3501922-supplementary.pdf]

Comparative study of biological (antioxidant, antimicrobial, antiprotozoal and cytotoxic) activity of some edible *Suillus* sp. fruiting body extracts

Asta Judžentienė and Jonas Šarlauskas

Table S1. Yields (%) obtained from *Suillus* sp. fruiting bodies by first (using methanol/water (1:1)) and second (with ethyl acetate) extraction procedures.

***Suillus* species**	**MeOH/H_2O**	**Ethyl acetate**
S. variegatus	21.66 ± 2.35	3.10 ± 0.42
S. luteus	18.88 ± 0.19	1.75 ± 0.22
S. bovinus	20.07 ± 0.33	2.09 ± 0.11
S. granulatus	17.02 ± 0.68	2.99 ± 0.18

Table S2. Effective concentration (EC_{50}, %) of various *Suillus* sp. extracts capable to scavenge 50% of DPPH•.

***Suillus* species**	**MeOH/H_2O**	**Ethyl acetate**
S. variegatus	2.65 ± 0.36	0.53 ± 0.10
S. luteus	2.23 ± 0.47	0.16 ± 0.05
S. bovinus	2.03 ± 0.40	0.70 ± 0.15
S. granulatus	0.80 ± 0.30	0.38 ± 0.05

Table S3. Effective concentration (EC50, %) of various *Suillus* sp. extracts capable to scavenge 50% of $ABTS^{\bullet +}$.

***Suillus* species**	**MeOH/H_2O**	**Ethyl acetate**
S. variegatus	1.49 ± 0.27	0.64 ± 0.16
S. luteus	2.05 ± 0.33	0.22 ± 0.05
S. bovinus	2.45 ± 0.39	1.75 ± 0.24
S. granulatus	0.95 ± 0.22	0.53 ± 0.05

Table S4. ABTS$^{•+}$ scavenging activity (mmol/L, expressed by TROLOX equivalent) of various *Suillus* sp. extracts.

***Suillus* species**	**MeOH/H_2O**	**Ethyl acetate**
S. variegatus	0.39 ± 0.07	0.73 ± 0.10
S. luteus	0.42 ± 0.10	0.68 ± 0.06
S. bovinus	0.35 ± 0.04	0.51 ± 0.08
S. granulatus	0.47 ± 0.03	0.26 ± 0.05
